# Accuracy of Nutrient Calculations Using the Consumer-Focused Online App MyFitnessPal: Validation Study

**DOI:** 10.2196/18237

**Published:** 2020-10-21

**Authors:** Charlotte Evenepoel, Egbert Clevers, Lise Deroover, Wendy Van Loo, Christophe Matthys, Kristin Verbeke

**Affiliations:** 1 Translational Research in Gastrointestinal Disorders Department of Chronic Diseases, Metabolism and Aging KU Leuven Leuven Belgium; 2 Nutrition & Obesity Unit, Clinical and Experimental Endocrinology Department of Chronic Diseases, Metabolism and Aging KU Leuven Leuven Belgium; 3 Department of Endocrinology University Hospitals Leuven KU Leuven Leuven Belgium; 4 Leuven Food Science and Nutrition Research Center KU Leuven Leuven Belgium

**Keywords:** dietary assessment, MyFitnessPal, Nubel, nutrition, online application, diet

## Abstract

**Background:**

Digital food registration via online platforms that are coupled to large food databases obviates the need for manual processing of dietary data. The reliability of such platforms depends on the quality of the associated food database.

**Objective:**

In this study, we validate the database of MyFitnessPal versus the Belgian food composition database, Nubel.

**Methods:**

After carefully given instructions, 50 participants used MyFitnessPal to each complete a 4-day dietary record 2 times (T1 and T2), with 1 month in between T1 and T2. Nutrient intake values were calculated either manually, using the food composition database Nubel, or automatically, using the database coupled to MyFitnessPal. First, nutrient values from T1 were used as a training set to develop an algorithm that defined upper limit values for energy intake, carbohydrates, fat, protein, fiber, sugar, cholesterol, and sodium. These limits were applied to the MyFitnessPal dataset extracted at T2 to remove extremely high and likely erroneous values. Original and cleaned T2 values were correlated with the Nubel calculated values. Bias was estimated using Bland-Altman plots. Finally, we simulated the impact of using MyFitnessPal for nutrient analysis instead of Nubel on the power of a study design that correlates nutrient intake to a chosen outcome variable.

**Results:**

Per food portion, the following upper limits were defined: 1500 kilocalories for total energy intake, 95 grams (g) for carbohydrates, 92 g for fat, 52 g for protein, 22 g for fiber, 70 g for sugar, 600 mg for cholesterol, and 3600 mg for sodium. Cleaning the dataset extracted at T2 resulted in a 2.8% rejection. Cleaned MyFitnessPal values demonstrated strong correlations with Nubel for energy intake (r=0.96), carbohydrates (r=0.90), fat (r=0.90), protein (r=0.90), fiber (r=0.80), and sugar (r=0.79), but weak correlations for cholesterol (ρ=0.51) and sodium (ρ=0.53); all *P* values were ≤.001. No bias was found between both methods, except for a fixed bias for fiber and a proportional bias for cholesterol. A 5-10% power loss should be taken into account when correlating energy intake and macronutrients obtained with MyFitnessPal to an outcome variable, compared to Nubel.

**Conclusions:**

Dietary analysis with MyFitnessPal is accurate and efficient for total energy intake, macronutrients, sugar, and fiber, but not for cholesterol and sodium.

## Introduction

Analysis of dietary records is an important part of nutritional and epidemiological research investigating the effects of diet on human health. Food composition tables are used to convert recorded food intake into nutrient intake. Individual food items from the dietary record are matched with an entry in the food composition table so that nutrient information can be extracted. This matching process is typically done manually and is therefore time- and labor-intensive, especially in large-scale studies or studies that require repeated dietary assessment over time [1].

Over the last years, a large number of mobile apps have been developed to track lifestyle habits such as physical activity, sleep, time management, and dietary intake. Such dietary intake digital platforms contain an online food database that immediately converts food items into nutrient values. With these online platforms, users electronically record their food intake and track the amount of calories and nutrients consumed. To do so, users select consumed food items from the associated food database or, in case an item is not registered, they add that item and the related nutritional information to the platform's database. Therefore, these databases are partially user-based. Generally, these platforms have been developed to promote weight loss by increasing the awareness of habits and progress through self-monitoring; however, they also appeal to nutritional research applications as well. Yet, the accuracy and reliability of these platforms rely on the quality of the associated databases.

The aim of this study is to evaluate the accuracy of nutrient intake values based on the database of MyFitnessPal [2]. MyFitnessPal is the most popular commercial nutrition weight loss app, with more than 165 million users in 2016 [3]. In the United States, 83% of the dietitians who participated in a survey held at the Food and Nutrition Conference and Expo 2015 recommended the use of nutrition and health-related apps, with MyFitnessPal and Fitbit mentioned most [4,5]. Furthermore, in a survey across the United Kingdom, Australia, Canada, New Zealand, and the United States, it was the preferred app for sports dietitians [6]. MyFitnessPal is characterized by its extensive and country-specific database of over 6 million food items and brands. This consumer app is user-friendly, contains a barcode scanner to rapidly add packed foods, and has both smartphone and web-based versions. In addition, the basic version of MyFitnessPal is freely available. We extracted values for energy intake and nutrient composition from dietary records entered in MyFitnessPal and compared them to the values calculated using the Belgian food composition database, Nubel [7].

## Methods

### Study Sample

This study used data from a previous intervention study that aimed to evaluate the impact of modified wheat bran on microbial fermentation and intestinal health [8]. We used 100 dietary records from 50 participants. Each participant used MyFitnessPal to complete a 4-day dietary record 2 times, with 1 month between the 2 records. These 2 time points generated 2 dietary intake datasets, referred to as T1 and T2. All participants used MyFitnessPal to record their dietary intake.

### Dietary Intake Assessment

Each participant received an account on MyFitnessPal and detailed instructions with illustrations about the use of MyFitnessPal. The manual included information on how to (1) select food items in the MyFitnessPal database, (2) indicate portion size, (3) register home recipes, and (4) use the favorites lists. To register packed food items, participants were instructed to scan the attached barcode or to select the corresponding item (correct brand) from the food item list within MyFitnessPal. If the item was not included in the list, participants were asked to enter the missing food item, including the nutritional data mentioned on the package. For generic food items such as bread, rice, pasta, fruits, and vegetables, we preregistered items in the MyFitnessPal database with the tag “Targid” (ie, the name of our research unit). These items contained nutritional information from the Belgian food composition database, Nubel. This preregistration of Targid-tagged food items was done for standardization purposes, as generic items often have multiple entries in MyFitnessPal with highly variable nutritional information. Participants were asked to preferentially select these items, and in case the searched item was not available in the Targid list, they were instructed to choose a green-flagged item. The green flag indicates that administrators of MyFitnessPal have revised the nutritional information. With regard to portion size, participants were instructed to weigh the consumed food items as much as possible, and when they were unable to do so, they were asked to select from listed portion sizes.

### Dietary Nutrient Quantification

Food items registered in MyFitnessPal were converted to nutrient values (ie, total energy intake, carbohydrates, fat, protein, sugars, fiber, sodium, and cholesterol), either using the MyFitnessPal database or using Nubel. The Nubel database contains the composition of 1194 basic food items, each product expressed per 100 grams. The MyFitnessPal food records are directly linked to the food composition data available in the MyFitnessPal app. Therefore, downloading the food records from the MyFitnessPal account immediately yields nutrient intake values. Conversion of the MyFitnessPal-registered food items to nutrient values using the Nubel database was performed according to a standard in-house procedure. Registered food items in MyFitnessPal with a food match in the Nubel database (eg, an apple, lasagne) were translated to nutrients using the Nubel food composition information. Homemade food without a match in the Nubel database (eg, Belgian endive with ham and cheese sauce) was translated to nutrients using the recipes described in a basic and comprehensive Belgian cookbook named *Ons Kookboek* [9]. For packed food, the nutritional information on the food label was used. If portion size was available in grams, nutrient values per consumed portion were calculated. Portion sizes defined in measurements (eg, “a slice of,” “a piece of”) were converted to grams using guidelines on the standardized quantification of food products [10].

### Data Cleaning

As MyFitnessPal is partially user-based and thus inherently prone to errors, we developed an algorithm that removes extremely high nutrient values very likely to be erroneous using Monte Carlo simulations [11]. Using the T1 dataset as a training set, we determined an upper limit of intake per food portion (using an in-house R script) for each nutrient. These limit values were obtained for each nutrient by iteratively increasing the putative limit value and including only intake values below the limit in the database (Multimedia Appendix 1). For each iteration, the correlation between the included Nubel and MyFitnessPal values was calculated. The nutrient intake value for which this correlation was maximal was defined as the upper limit for that nutrient. These cut-offs constitute the best compromise between removing erroneous values (ie, true positives) and not removing correct values (ie, false positives). Subsequently, these limit values based on the T1 dataset were applied to the MyFitnessPal values of the T2 dataset to remove extremely high values, using an in-house R script (Multimedia Appendix 2).

### Data Analysis

For each 4-day diary, mean energy intake and nutrient values were calculated with Nubel and the MyFitnessPal database. To evaluate the impact of the data cleaning procedure, both the original and cleaned T2 data from MyFitnessPal were correlated to the Nubel values. The normality of the data was checked with the Shapiro-Wilk test and with visual inspection of the residual histogram. Depending on normality, Pearson or Spearman rank were applied for correlation analysis. Bland-Altman plots were used to assess the degree of agreement between both methods and to evaluate bias [12]. In addition, a paired *t* test or a Wilcoxon signed rank test was performed. Data analysis was performed using SAS software (version 9.4; SAS Institute). *P* values less than .05 were considered statistically significant.

To further assess the practical implication of using the MyFitnessPal database, we simulated the loss of statistical power that occurs when using the MyFitnessPal database compared to the Nubel database in a study design that aims to correlate nutrient intake to a random outcome variable. In addition, we calculated the corresponding increase in sample size required to compensate for the loss in power. Given a nutrient quantified using Nubel and MyFitnessPal, named N and M, respectively, and given a variable defined as the outcome (O), we used the correlation NM (known from this study) and a simulated correlation NO to retrieve the correlation of interest (ie, MO). Simulations were performed according to Monte Carlo simulations [11]. Briefly, for each nutrient, we simulated 100 times a value for O and this for 100 correlations, with 0≤ ρ ≤ 0.5 and a step length of 0.005. Subsequently, for each correlation between O and the Nubel-calculated nutrient N, and with a known sample size, we derived the power for NO. Corresponding correlations between the O variables and the MyFitnessPal-calculated nutrients M were computed, followed by the resulting power of MO. The power of MO indicates the power to detect a true effect for the correlation of the MyFitnessPal nutrient data (M) and the outcome variable (O) (eg, a health parameter). This simulation was performed for a sample size of 50, 100, and 500 observations. All simulation analyses were performed with R (version 3.5.1; R Core Team). Power calculations were performed with R package “pwr” (version 1.3-0; Stéphane Champely).

## Results

Of the 50 participants, 78% (39/50) were women and 22% (11/50) were men. Mean BMI was 25.3 (SD 5.1) kg/m^2^ and mean age was 28.2 (SD 11.3) years. All dietary records covered 4 consecutive days, except for 1 diary with only 3 days of food registration. The upper limit values per food portion were 1500 kilocalories (kcal) for total energy intake, 95 grams (g) for carbohydrates, 92 g for fat, 52 g for protein, 22 g for fiber, 70 g for sugar, 600 mg for cholesterol, and 3600 mg for sodium. The clean-up of dataset T2 removed certain nutrient values from 79 of the 2826 recorded food items (2.8%). For carbohydrates, 46 values were removed; for protein, 17 values were removed; for fat, 2 values were removed; for sugar, 8 values were removed; for cholesterol and sodium, 3 values were removed. No values for fiber and total energy intake were removed.

In the original T2 dataset, strong positive correlations between Nubel and MyFitnessPal were obtained for total energy intake, the 3 macronutrients, and for sugar and fiber (Table 1). Nevertheless, fiber intake was significantly underestimated when calculated with the MyFitnessPal database (*P*<.001). Correlations for cholesterol and sodium, however, were weak, and their intake was also strongly underestimated by MyFitnessPal. After the cleaning of the T2 dataset, correlations with Nubel data were stronger for carbohydrates, fat, sugar, and sodium than for the original data (Table 1). Mean energy and fat intake did not differ significantly between cleaned MyFitnessPal and Nubel values, whereas carbohydrate and protein intake became slightly but significantly lower using MyFitnessPal. Correlations for original and cleaned T2 MyFitnessPal data with Nubel were identical for fiber and total energy intake, as data cleaning did not remove any value.

**Figure 1:**
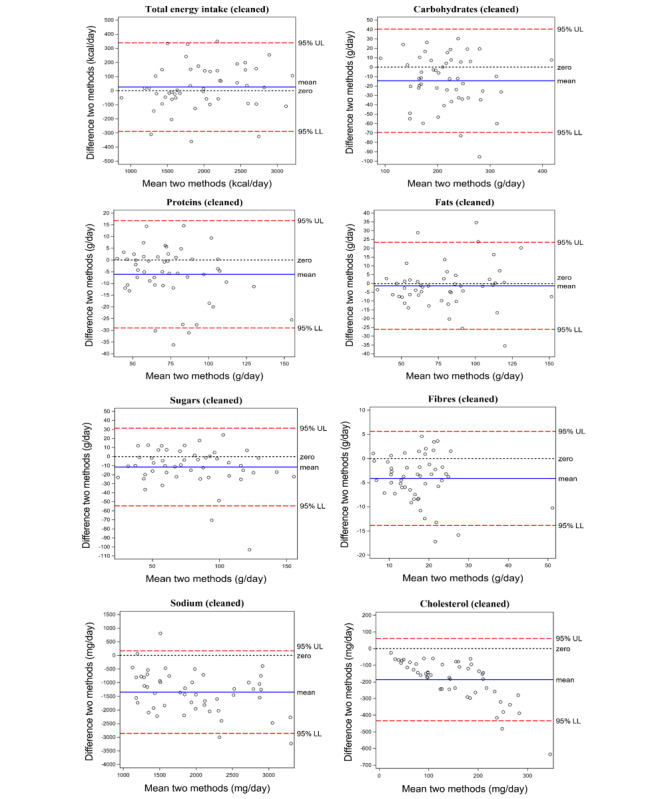
Bland-Altman plots for energy intake and nutrient values of the cleaned T2 dataset, with Nubel as the reference method and MyFitnessPal as the other method for nutrient intake analysis. The difference between the 2 methods is calculated as follows: MyFitnessPal – Nubel. The 95% upper limit (UL) and lower limit (LL) of agreement (SD 1.96) are depicted as long dashed lines. The full line and short-dashed line indicate the mean difference and zero, respectively.

Bland-Altman plots of the cleaned T2 data displaying the agreement between both methods for all nutrients and energy intake are shown in Figure 1. No proportional nor fixed bias was observed for the macronutrients, sugar, and energy intake. In contrast, fiber intake showed an average fixed bias of about 4 g/day, which is about 20% of average fiber intake. Furthermore, sodium and cholesterol intake were clearly underreported in MyFitnessPal [the mean difference MyFitnessPal-Nubel amounts to ‑1345 (SD 241) mg/day for sodium and ‑187 (SD 124) mg/day for cholesterol]. In addition, the difference between MyFitnessPal and Nubel for cholesterol intake proportionally increased with increasing cholesterol intake.

**Figure 2:**
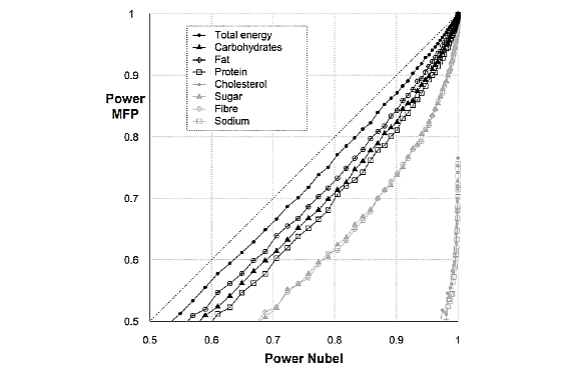
Correlation analysis between the statistical power of Nubel and MyFitnessPal (MFP) to reject the null hypothesis that states there is no correlation between each of these methods and a simulated variable outcome. The total sample size is 100 power values. This correlation between the power of Nubel and MyFitnessPal was performed for all studied nutrients (macronutrients, sugar, fiber, cholesterol, and sodium) and for energy intake.

The power simulation quantified the statistical power that is lost when correlating MyFitnessPal-derived data instead of Nubel data to an outcome parameter (Figure 2) and the additional sample size needed to compensate (Table 2). For example, for an intended power of 80% with Nubel, the power will be decreased when using MyFitnessPal with 4% for total energy intake, 8% for carbohydrates, 10% for protein and fat, 15% for sugar, and 19% for fiber. Depending on the intended sample size, the required increase in sample size to compensate for this loss in power ranged from 10% to 65% (Table 2). Furthermore, the simulation showed a complete loss of power if MyFitnessPal would be used to assess cholesterol and sodium intake, resulting in extremely high sample sizes.

**Table 1:** Nutrient intake values of T2 data derived from Nubel and MyFitnessPal, prior to and after data cleaning (n=50).

Nutrient	Nubel		Original MyFitnessPal data				Cleaned MyFitnessPal data			
	Mean	SD	Mean	SD	*P* value^a^	Coefficient^b^	Mean	SD	*P* value^a^	Coefficient^b^
Energy intake (kcal/day)	1958	543	1984	567	.26	0.96	1984	567	.26	0.96
Carbohydrates (g/day)	225	61	241	80	.05	0.70	210	58	<.001	0.90
Fat (g/day)	81	27	82	31	.69	0.75	80	28	.44	0.90
Protein (g/day)	78	26	78	26	.85	0.94	72	23	<.001	0.90
Sugar (g/day)	85	35	80	40	.22	0.70	74	31	<.001	0.79
Fiber (g/day)	19	8	15	7	<.001	0.80	15	7	<.001	0.80
Sodium (mg/day)	2638	848	1551	1765	<.001	0.45	1293	607	<.001	0.53
Cholesterol (mg/day)	242	131	67	63	<.001	0.67	55	49	<.001	0.51

^a^Paired *t* test was used for energy intake, macronutrients, sugar, and fiber; the Wilcoxon signed rank test was used for for cholesterol and sodium.

^b^Pearson correlation coefficients were used for energy intake, macronutrients, sugar, and fiber; the Spearman rank correlation coefficients were used for cholesterol and sodium. Both Pearson and Spearman were significant at the level of .05, as they were all *P*<.001.

**Table 2:** Increase in sample size (%) required to maintain 80% power when using MyFitnessPal for nutrient analysis instead of Nubel to detect a true effect if correlated with an outcome variable.

Nubel sample size	Percentage (%) increase in MyFitnessPal sample size required to maintain a statistical power of 80%							
	Energy intake	Carbohydrate	Fat	Protein	Sugar	Fiber	Cholesterol	Sodium
50	11	28	21	33	64	65	347	364
100	10	27	20	32	60	65	277	307
500	10	25	19	28	40	36	68	72

## Discussion

### Principal Findings

This study evaluated the accuracy of the MyFitnessPal food composition database to assess energy and macro- and micronutrient intake. Strong positive correlations between MyFitnessPal and Nubel were observed for energy intake, macronutrients, sugar, and fiber, but not for cholesterol and sodium. Bland-Altman plots displayed a good agreement between MyFitnessPal and Nubel, except for cholesterol and sodium. Using MyFitnessPal over Nubel for quantifying total energy intake and macronutrients led to a power loss in studies that correlate nutrient values to an outcome variable of not more than 10%, which can be compensated by increasing the sample size.

Digital collection of food intake data facilitates the assessment of dietary intake, both at the level of dietary data registration and at the level of nutrient quantification [1,13]. Many studies have compared digital methods with paper-and-pencil methods at the level of dietary registration using the same database for nutrient calculation. These studies, using digital registration tools such as My Meal Mate, YANA-C, Wellnavi, e-DIA or Easy Diet Diary, observed good agreement between the digital and paper method [14-18]. In this study, we used the same digital method for diet registration but applied 2 distinct databases to calculate the nutrient intake values. Our results indicate that the nutritional information extracted from MyFitnessPal is comparable to the information calculated with a standard food composition database (Nubel), except for cholesterol and sodium. Overall, dietary intake was slightly underestimated by MyFitnessPal, which is in agreement with previous studies [19,20]. The underestimation was even more pronounced after cleaning the dataset, a procedure that resulted in the rejection of extremely high and likely erroneous values. Compared to Nubel-derived values, MyFitnessPal underestimated protein intake by 7.8%, carbohydrate intake by 6.4%, and fat intake by 1.7%. In contrast, energy intake was slightly overestimated (1.3%). In a study applying the Brazilian food composition data table as a reference, the MyFitnessPal database underestimated energy (0.7%), fat (16.8%), protein (11.9%), and carbohydrate intake (10.8%) [19]. In another study, nutrient intake estimates from thirty 24-hour dietary recalls collected using the Nutrition Data System for Research (NDSR) were compared with intake calculations from these recalls entered by the researcher into MyFitnessPal [20]. Compared to NDSR, a similar underestimation by MyFitnessPal was observed for energy (4.1%), fat (14.1%), and protein (8.0%), while carbohydrate intake was overestimated by 1.6% [20]. A reason for the underestimation by MyFitnessPal is most likely incomplete or missing information about nutrient composition for some food items in the database. Indeed, some entries in MyFitnessPal only have a value for total energy content without values for macronutrient composition or cholesterol and sodium content. Selecting such items for inclusion in the dietary record results in inaccurate information.

The use of digital platforms like MyFitnessPal has several advantages over other methods. Platforms available as a smartphone app allow participants to report food intake conveniently on their smartphones, regardless of their whereabouts or the occasion. In this way, the time delay between consuming and registering food data is reduced, which increases data quality [21]. Compared to food frequency questionnaires and 24-hour recalls, prospective food recording is less prone to memory bias [22,23]. Additionally, recording with a mobile app increases the satisfaction and adherence of the participants, further enhancing dietary assessment [24,25]. Moreover, the direct link of the consumed items to the nutritional facts facilitates and accelerates the work of the investigator. In contrast to paper data, MyFitnessPal food data do not require transfer to an electronic database, which reduces errors. In this way, the use of MyFitnessPal can reduce the high financial and human resources required for standard nutrient quantification [26]. A potential concern of digital platforms is the fact that the immediate feedback and nutritional information provided to participants may alter their eating behavior and induce misreporting. This is called the “reactivity effect” and should be taken into consideration when deciding on the use of a digital platform in a study design [27].

An important source of error in dietary assessment, independent of the registration method and database used, is the estimation of the portion size by the participant. A study that compared meals recorded by the participants with a personal digital assistant (PDA) against actual meals assessed by dietitians reported portion size to be the greatest source of error, accounting for 49% of the errors between recorded and actual meals [28]. Other major errors in food registration by participants included reporting incorrect food (25%) and omitting food (15%). In addition, in a study with the digital My Meal Mate diary, an incorrect portion size was the cause of most errors, with users selecting the standard listed portion sizes rather than providing the true portions consumed [14].

An effective strategy to improve the accuracy of dietary assessment with digital platforms is to provide adequate training to the participants, in the form of a manual or a training session. A study of 78 adolescents showed a significant increase in proficiency and perception in the use of a mobile phone food record after additional training was provided [29]. Accordingly, the application of MyFitnessPal in a naturalistic setting (ie, without the provision of instructions to subjects unfamiliar with the app) resulted in poor correlations for 4-day mean energy and macronutrient intake (ranging from 0.21 to 0.42) compared to a 24-hour recall analyzed with the Australian Food, Supplement, and Nutrient Database (AUSNUT) 2011-2013 database [30]. Therefore, it is crucial that the training includes information on how to select items from the database and clear instructions on how to provide portion sizes. We assume that the high correlations between MyFitnessPal and Nubel found in this study are partly due to the extensive manual that was provided to the participants and the fact that we predefined a number of generic items to increase standardization. In daily life, this manual is not available, and as MyFitnessPal is a user-based platform, errors in nutritional information arise in the database. However, MyFitnessPal itself has built-in tools to enhance food registration, such as bar code scanning and the green flagging of items. If the user is motivated, MyFitnessPal may be a useful tool to track nutrient intake in daily life. Using MyFitnessPal as a self-guided approach was as effective in inducing weight loss as a combination of the app with dietary counseling in 100 obese subjects [31]. Another trial highlighted the importance of motivation when using MyFitnessPal for weight loss management in combination with dietary counseling [25]. In addition, it is important to realize that our study was performed in the context of a trial evaluating the health benefits of wheat bran. Therefore, participants were likely biased towards persons interested in human health. Good motivation and background knowledge on nutrition and food are known predictors for reliable and accurate reporting with digital dietary records [22]. Consequently, these predictors may have driven our study participants to select those food items with accurate nutritional information and may have directed our results.

### Conclusion

MyFitnessPal provides accurate estimates for energy, macronutrients, fiber, and sugar intake, but not for cholesterol and sodium, in a research setting. We advise the use of both a data cleaning procedure and a clear manual on dietary reporting for participants, as they contribute to better nutrient assessment. A manual on MyFitnessPal usage should include instructions on proper food item selection, portion size recording, and the use of features such as scanning bar codes of food items. To enhance standardization, it is useful to add entries with a tag for generic items, as the MyFitnessPal database contains many entries with considerable differences in nutritional information.

## Appendix

Multimedia Appendix 1 Flow chart representing the iterative process that was applied to define the upper limit for a nutrient intake value extracted from MyFitnessPal (MFP) using Monte Carlo simulations.

Multimedia Appendix 2 In-house R script for nutrient data extraction from MyFitnessPal, including data cleaning.

## Abbreviations

AUSNUT: Australian Food, Supplement, and Nutrient Database
g: grams
kcal: kilocalories
NDSR: Nutrition Data System for Research
PDA: personal digital assistant
