# Supplementary figures and images for: Accuracy of Nutrient Calculations Using the Consumer-Focused Online App MyFitnessPal: Validation Study
Source: J Med Internet Res. 2020 Oct 21;22(10):e18237. doi: 10.2196/18237 (PMC7641788; doi:10.2196/18237)

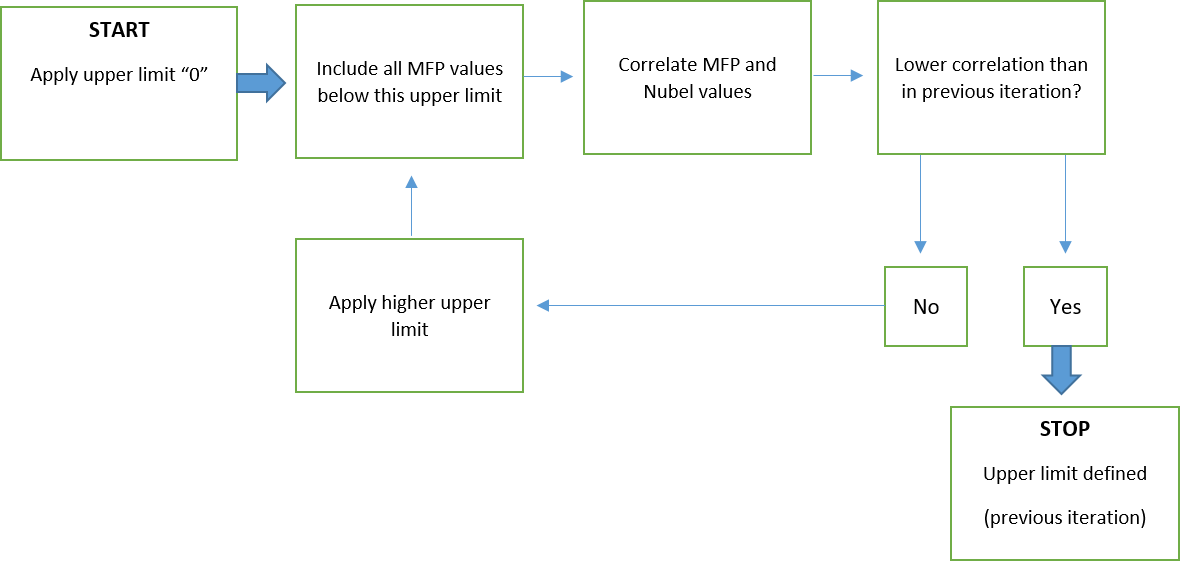

Supplement: Multimedia Appendix 1 [file jmir_v22i10e18237_app1.png]
