# Supplementary material for: Accuracy of Nutrient Calculations Using the Consumer-Focused Online App MyFitnessPal: Validation Study
Source: J Med Internet Res. 2020 Oct 21;22(10):e18237. doi: 10.2196/18237 (PMC7641788; doi:10.2196/18237)
Supplement: Multimedia Appendix 2 [file jmir_v22i10e18237_app2.docx]

**Multimedia Appendix 2**. In-house R script for nutrient data extraction from MyFitnessPal, including data cleaning.

# Script to extract and clean nutrient data from MyFitnessPal, by Egbert Clevers

# For questions, please contact me via LinkedIn

# The below 3 lines must be adapted.

LocationOfTxtFiles<-"J: /Txt files"

LocationOfNormalisedFiles<-"J: /Normalised files"

RemoveNutrientsNotPassingQC<-TRUE

# The below are data-driven cut-offs for requiring data cleaning.

Nutrients<-c("kCal","Carbohydrates","Fats","Proteins","Cholesterol","Sodium","Sugars","Fibres")

QClimits<-rep(NA,8)

names(QClimits)<-Nutrients

QClimits[1]<-1500 # kCal

QClimits[2]<-95 # Carbohydrates (g)

QClimits[3]<-92 # Fats (g)

QClimits[4]<-52 # Proteins (g)

QClimits[5]<-600 # Cholesterol (mg)

QClimits[6]<-3600 # Sodium (mg)

QClimits[7]<-70 # Sugars (g)

QClimits[8]<-22 # Fibres (g)

MultiplierForAutomatedRemoval<-1

# install.packages("matrixStats")

# install.packages("xlsx")

# install.packages("stringi")

# install.packages("stringr")

library(matrixStats)

library(xlsx)

library(stringi)

library(stringr)

setwd(LocationOfTxtFiles)

FileNames<-list.files()

FileNames<-FileNames[regexpr(".txt",FileNames)>1]

Participants<-str_split(FileNames,".txt",simplify=T)[,1]

SummaryTable<-matrix(rep(NA,length(FileNames)*9),ncol=9)

rownames(SummaryTable)<-Participants

colnames(SummaryTable)<-c("Days",Nutrients)

Units<-list(c("kilogr","kg"," kilo"," liter"," ltr"," litre"),c("hgr"," hg","hectog")," mg",c(" dgr"," cl"," cc"),c(" gr"," gm","ml"," millil"))

# Loops over the txt files

for(a in 1:length(FileNames))

{

setwd(LocationOfTxtFiles)

# Loads the file

DataFile<-as.character(read.delim(FileNames[a],header=F)[,1])

DataFile<-gsub("--mg","0mg",DataFile,fixed=T)

DataFile<-gsub("--g","0g",DataFile,fixed=T)

DataFile<-gsub("--m","0mg",DataFile,fixed=T)

Counter=Counter2=CurrentDate=0

Language<-"Unknown"

RemoveFromMean<-rep(0,8)

Listen=SkipNext=Escape=FALSE

NormalisedFile<-data.frame()

# ---------------------------------

# Removes spaces at the start of a string (which are often there following conversion from PDF to txt).

for(b in 1:length(DataFile))

{

SpacesInString<-stri_locate_all(pattern=" ",DataFile[b],fixed=TRUE)[[1]]

if(length(SpacesInString)>0)

{

if(SpacesInString[1,1]==1)

{

SpacesInString<-sapply(1:max(SpacesInString[,1]),function(c){if(c%in%SpacesInString[,1]){1}else{0}})

SpacesInString<-rle(SpacesInString)$lengths[1]

DataFile[b]<-str_sub(DataFile[b],SpacesInString+1,nchar(DataFile[b]))

}

}

}

NumberOfDays<-sum(sapply(1:length(DataFile),function(b){(nchar(str_sub(DataFile[b],1,13))-min(nchar(sapply(1:12,function(c){gsub(paste(month.name[c],"",collapse=""),"",DataFile[b])})))>0|nchar(str_sub(DataFile[b],1,13))-min(nchar(sapply(1:12,function(c){gsub(paste(c("januari","februari","maart","april","mei","juni","juli","augustus","september","oktober","november","december")[c],"",collapse=""),"",DataFile[b])})))>0)&nchar(DataFile[b])-min(nchar(sapply(1:41,function(c){gsub(c(2010:2050)[c],"",DataFile[b])})))>0}))

# ---------------------------------

DataFile<-c(DataFile," ")

# Loops over all the lines of the file, aiming to extract food intake data.

for(b in 1:length(DataFile))

{

if(str_sub(DataFile[b],1,8)%in%c("EXERCISE","Food Not","Aanteken","ACTIVITE")){Listen<-FALSE}

# SkipNext applies for long food strings that cover 2 lines in the PDF file.

if(SkipNext==TRUE|Counter2>0)

{

SkipNext<-FALSE

Counter2<-Counter2-1

next

}

# ---------------------------------

# Adds the day totals to the normalised file. Does so whenever a new date is encountered, or at the end of the file.

if((nchar(str_sub(DataFile[b],1,13))-min(nchar(sapply(1:12,function(c){gsub(paste(month.name[c],"",collapse=""),"",DataFile[b])})))>0|nchar(str_sub(DataFile[b],1,13))-min(nchar(sapply(1:12,function(c){gsub(paste(c("januari","februari","maart","april","mei","juni","juli","augustus","september","oktober","november","december")[c],"",collapse=""),"",DataFile[b])})))>0)&nchar(DataFile[b])-min(nchar(sapply(1:41,function(c){gsub(c(2010:2050)[c],"",DataFile[b])})))>0|b==length(DataFile))

{

# Gets language.

if(nchar(str_sub(DataFile[b],1,13))-min(nchar(sapply(1:12,function(c){gsub(paste(c("januari","februari","maart","april","mei","juni","juli","augustus","september","oktober","november","december")[c],"",collapse=""),"",DataFile[b])})))>0&nchar(DataFile[b])-min(nchar(sapply(1:41,function(c){gsub(c(2010:2050)[c],"",DataFile[b])})))>0|b==length(DataFile)){Language="Nederlands"}else{Language="English"}

if(Counter>1)

{

Counter<-Counter+1

for(c in 1:8){NormalisedFile[Counter,c+4]<-TotalNutrients[c]}

NormalisedFile[Counter,1:4]<-c("","DAY TOTAL","","")

}

if(b==length(DataFile)){next}

CurrentDate<-DataFile[b]

NewDate<-TRUE

Listen<-FALSE

next

}

# ---------------------------------

if(DataFile[b]%in%c("Breakfast","Lunch","Dinner","Snacks","Ontbijt","Middageten","Avondeten","Tussendoortjes")==TRUE)

{

Listen<-TRUE

next

}

# Code to fetch daily nutrient sums from the txt file.

if(str_sub(DataFile[b],1,5)%in%c("TOTAL","TOTAA")&!str_sub(DataFile[b],1,6)=="TOTALS"&!str_sub(DataFile[b],1,7)=="TOTALEN")

{

SpacesInString<-stri_locate_all(pattern=" ",DataFile[b],fixed=TRUE)[[1]]

SpacesInString<-sapply(1:nchar(DataFile[b]),function(c){if(c%in%SpacesInString[,1]){1}else{0}})

TotalNutrients<-paste(" ",str_sub(DataFile[b],max(cumsum(head(rle(SpacesInString)$lengths,length(rle(SpacesInString)$lengths)-15))),nchar(DataFile[b])),collapse="")

SpacesInString<-stri_locate_all(pattern=" ",TotalNutrients,fixed=TRUE)[[1]]

SpacesInString<-sapply(1:nchar(TotalNutrients),function(c){if(c%in%SpacesInString[,1]){1}else{0}})

TotalNutrients<-sapply(1:8,function(c){str_sub(TotalNutrients,cumsum(rle(SpacesInString)$lengths)[c*2-1],cumsum(rle(SpacesInString)$lengths)[c*2])})

}

# Identifies a food entry.

if(Listen==TRUE&!str_sub(DataFile[b],1,5)%in%c(paste0(1:9," van"),paste0(10:20," va"),"TOTAL","TOTAA","FOODS","VOEDI","http:","https")&!str_sub(DataFile[b],1,2)%in%c("/f","\f","\fP")&!str_sub(DataFile[b],1,1)%in%c("/f","\f"))

{

SpacesInString<-stri_locate_all(pattern=" ",DataFile[b],fixed=TRUE)[[1]]

if(length(SpacesInString)>0)

{

SpacesInString<-sapply(1:nchar(DataFile[b]),function(c){if(c%in%SpacesInString[,1]){1}else{0}})

}

if(rle(SpacesInString)$lengths[1]<5&rle(SpacesInString)$lengths[2]>5){next}

Counter<-Counter+1

# Get food item name.

CurrentItem<-str_split(DataFile[b]," ",simplify=T)[1,1]

# Detects that nutrient values are in the string.

if(length(rle(SpacesInString)$lengths)>16&nrow(stri_locate_all(pattern="g",DataFile[b],fixed=TRUE)[[1]])>5&nchar(gsub("[^\\d]+","",DataFile[b],perl=TRUE))>6)

{

# Attempts to split the item name from the nutrient values.

CurrentItem<-str_sub(DataFile[b],1,max(cumsum(head(rle(SpacesInString)$lengths,length(rle(SpacesInString)$lengths)-16))))

# Gets the nutrient values.

CurrentNutrients<-paste(" ",str_sub(DataFile[b],max(cumsum(head(rle(SpacesInString)$lengths,length(rle(SpacesInString)$lengths)-15))),nchar(DataFile[b])),collapse="")

}

if(b<length(DataFile)-1)

{

# Detects that the current item is a long string that covers 2 lines.

SkipNext<-if(nchar(gsub("[^\\d]+","",DataFile[b],perl=TRUE))<8&nchar(CurrentItem)>20&nrow(stri_locate_all(pattern="g",DataFile[b+1],fixed=TRUE)[[1]])>5&(nrow(stri_locate_all(pattern="g",DataFile[b+2],fixed=TRUE)[[1]])<5|nchar(gsub("[^\\d]+","",DataFile[b+2],perl=TRUE))<8)&nchar(DataFile[b+2])-nchar(gsub("-","",DataFile[b+2]))==0&!DataFile[b+1]%in%c("Breakfast","Lunch","Dinner","Snacks","Ontbijt","Middageten","Avondeten","Tussendoortjes")&!DataFile[b+2]%in%c("Breakfast","Lunch","Dinner","Snacks","Ontbijt","Middageten","Avondeten","Tussendoortjes")&(nchar(str_sub(DataFile[b+2],1,10))-min(nchar(sapply(1:12,function(c){gsub(paste(month.name[c],"",collapse=""),"",DataFile[b+2])})))==0|nchar(DataFile[b+2])-min(nchar(sapply(1:5,function(c){gsub(c(2015:2019)[c],"",DataFile[b+2])})))==0)&!str_sub(DataFile[b+1],1,5)%in%c("TOTAL","TOTAA","FOODS","VOEDI","http:")){TRUE}else{FALSE}

if(nchar(DataFile[b])-nchar(gsub("Mini Ribbetjes Picante (Merk: Tapas Club","",DataFile[b],fixed=TRUE))>0){SkipNext<-TRUE}

if(nchar(DataFile[b])-nchar(gsub("Atelier Louis - Abrikoos-frangipanetaart, Tarte","",DataFile[b],fixed=TRUE))>0){SkipNext<-TRUE}

if(SkipNext==TRUE)

{

# In case the txt file is wrongly formatted, escapes and print a warning message.

if(nchar(DataFile[b+1])>180)

{

Escape<-TRUE

print(paste0("The file ",FileNames[a]," was not correctly loaded. It may contain a quotation mark (or similar symbol) around line ",b+1,". Please try removing this symbol in the source txt file. Moving on to the next file for now..."))

break

}

# Combines the string that covers 2 lines so that it is one unit.

CurrentItem<-paste(c(CurrentItem,DataFile[b+2]),collapse=" ")

CurrentNutrients<-paste(" ",DataFile[b+1],collapse="")

Counter2<-2

}

}

# Splits the product name from the quantity (based on the rightmost comma).

CurrentProduct<-str_sub(CurrentItem,1,tail(gregexpr(",",CurrentItem)[[1]],1)-1)

CurrentQuantity<-str_sub(CurrentItem,tail(gregexpr(",",CurrentItem)[[1]],1)+1,nchar(CurrentItem))

# Gets rid of 1000-indicators ("." in Dutch, "," in English), and homogenises decimal indicators.

CurrentNutrients<-gsub(if(Language=="Nederlands"){"."}else{","},"",CurrentNutrients,fixed=T)

if(Language=="Nederlands"){CurrentNutrients<-gsub(",",".",CurrentNutrients,fixed=T)}

SpacesInString<-stri_locate_all(pattern=" ",CurrentNutrients,fixed=TRUE)[[1]]

if(length(SpacesInString)>0)

{

SpacesInString<-sapply(1:nchar(CurrentNutrients),function(c){if(c%in%SpacesInString[,1]){1}else{0}})

CumSum<-cumsum(rle(SpacesInString)$lengths)

Length<-length(CumSum)

Start<-max(c(1:Length)[c(1:Length)%%2==1])-(8*2)

CurrentNutrients<-sapply(1:8,function(c){str_sub(CurrentNutrients,CumSum[Start+c*2],CumSum[Start+c*2+1])})

}

# Inserts the food product and nutrient values into the normalised file.

NormalisedFile[Counter,1]<-if(NewDate==FALSE){""}else{CurrentDate}

NormalisedFile[Counter,2]<-if(nchar(DataFile[b])-nchar(gsub("-","",DataFile[b]))==0){"(Brand unknown)"}else{str_sub(CurrentProduct,1,tail(gregexpr("-",CurrentProduct)[[1]],1)-1)}

NormalisedFile[Counter,3]<-str_sub(CurrentProduct,tail(gregexpr("-",CurrentProduct)[[1]],1)+1,nchar(CurrentProduct))

NormalisedFile[Counter,4]<-gsub(if(Language=="Nederlands"){"."}else{","},"",CurrentQuantity,fixed=T)

for(c in 1:8){NormalisedFile[Counter,c+4]<-CurrentNutrients[c]}

NewDate<-FALSE

# ---------------------------------

# Data cleaning of nutrient values. Starts by extracting the quantity in grams where available.

Quantity<-tolower(NormalisedFile[Counter,4])

Success<-FALSE

for(Type in 1:5)

{

for(Unit in Units[[Type]])

{

if(nchar(Quantity)-nchar(gsub(Unit,"",Quantity))>0)

{

String<-Unit

StringLocation<-str_locate(Quantity,String)[1,]

Matches<-unlist(regmatches(Quantity,gregexpr("[[:digit:]]+",Quantity)))

NumberLocation<-cbind(sapply(Matches,function(a){str_locate(Quantity,a)[1,]}))

Value<-0

if(ncol(NumberLocation)==2){if(NumberLocation[1,2]-NumberLocation[2,1]==2){Value<-as.numeric(paste0(Matches[1],".",Matches[2]))}}

if(Value==0&length(Matches)==1){Value<-as.numeric(Matches)}

if(Value==0){break}

Value<-Value*(c(1000,100,0.001,10,1)[Type])

if(Type==1){while(Value>2000){Value<-Value/10}}

Success<-TRUE

break

}

}

if(Success==TRUE){break}

}

if(Success==FALSE&str_sub(Quantity,nchar(Quantity)-1,nchar(Quantity))==" g")

{

Matches<-unlist(regmatches(Quantity,gregexpr("[[:digit:]]+",Quantity)))

NumberLocation<-cbind(sapply(Matches,function(a){str_locate(Quantity,a)[1,]}))

Value<-0

if(ncol(NumberLocation)==2){if(NumberLocation[1,2]-NumberLocation[2,1]==2){Value<-as.numeric(paste0(Matches[1],".",Matches[2]))}}

if(Value==0&length(Matches)==1){Value<-as.numeric(Matches)}

if(Value!=0)

{

Unit<-" gr"

Success<-TRUE

}

}

if(Success==FALSE)

{

Value<-NA

Unit<-NA

}

ExistingNutrients<-NormalisedFile[Counter,5:12]

for(c in 1:27){ExistingNutrients<-gsub(c(letters," ")[c],"",ExistingNutrients)}

ExistingNutrients<-as.numeric(ExistingNutrients)

TotalDryWeight<-if(is.na(Value)){NA}else{sum(c(ExistingNutrients[c(2:4)],ExistingNutrients[5:6]/1000),na.rm=T)}

Flags<-0

NormalisedFile[Counter,13]<-""

NormalisedFile[Counter,14]<-0

NormalisedFile[Counter,15]<-""

# Removal nutrient value when above upper limit.

for(c in 1:8)

{

if(ExistingNutrients[c]>QClimits[c])

{

NormalisedFile[Counter,13]<-paste0(NormalisedFile[Counter,13]," / ",paste0("High ",Nutrients[c]))

Flags<-Flags+1

if(RemoveNutrientsNotPassingQC==TRUE&ExistingNutrients[c]>(QClimits[c]*MultiplierForAutomatedRemoval))

{

RemoveFromMean[c]<-RemoveFromMean[c]+ExistingNutrients[c]/NumberOfDays

NormalisedFile[Counter,15]<-if(nchar(NormalisedFile[Counter,15])<3){Nutrients[c]}else{paste0(NormalisedFile[Counter,15],", ",Nutrients[c])}

}

}

}

NormalisedFile[Counter,14]<-Flags

# ---------------------------------

}

}

colnames(NormalisedFile)<-c("Date","Brand","Product","Quantity",Nutrients,"Item.needs.check","Flags","Removed")

for(Column in 13:15){NormalisedFile[is.na(NormalisedFile[,Column]),Column]<-""}

assign(paste0("PP",Participants[a]),NormalisedFile)

setwd(LocationOfNormalisedFiles)

# Gets number of days.

SummaryTable[a,1]<-sum(NormalisedFile[,1]!="")

if(Escape==FALSE)

{

for(b in 5:12)

{

DayTotals<-NormalisedFile[NormalisedFile[,"Brand"]=="DAY TOTAL",b]

DayTotals<-gsub(if(Language=="Nederlands"){"."}else{","},"",DayTotals,fixed=T)

DayTotals<-gsub(",","",DayTotals,fixed=T)

DayTotals<-gsub(".","",DayTotals,fixed=T)

NormalisedFile[NormalisedFile[,"Brand"]=="DAY TOTAL",b]<-DayTotals

for(c in 1:27){DayTotals<-gsub(c(letters," ")[c],"",DayTotals)}

DayTotals<-as.numeric(DayTotals)

SummaryTable[a,b-3]<-mean(DayTotals,na.rm=T)-RemoveFromMean[b-4]

}

}

# Writes the normalised file to the disk.

write.xlsx(NormalisedFile,paste0(if(Escape==TRUE){"ERROR."}else{"PP."},Participants[a],".xlsx"),row.names=F)

rm(a,b,c,Counter,Counter2,CurrentDate,CurrentItem,CurrentNutrients,CurrentProduct,CurrentQuantity,DataFile,Listen,NewDate,NormalisedFile,SkipNext,SpacesInString,TotalNutrients)

}

rm(FileNames)

setwd(LocationOfNormalisedFiles)

write.table(SummaryTable,"SummaryTable.txt",sep="\t",dec=",")
